# Lung Ultrasound in the Management of Acute Decompensated Heart Failure

**DOI:** 10.2174/157340312801784907

**Published:** 2012-05

**Authors:** Shiang-Hu Ang, Phillip Andrus

**Affiliations:** 1 Department of Emergency Medicine, Changi General Hospital, Singapore; 2 Department of Emergency Medicine, Mount Sinai School of Medicine, New York, NY. USA

**Keywords:** Ultrasonography, lung, management, acute decompensated heart failure.

## Abstract

Once thought impracticable, lung ultrasound is now used in patients with a variety of pulmonary processes. This review seeks to describe the utility of lung ultrasound in the management of patients with acute decompensated heart failure (ADHF). A literature search was carried out on PubMed/Medline using search terms related to the topic. Over three thousand results were narrowed down via title and/or abstract review. Related articles were downloaded for full review. Case reports, letters, reviews and editorials were excluded. Lung ultrasonographic multiple B-lines are a good indicator of alveolar interstitial syndrome but are not specific for ADHF. The absence of multiple B-lines can be used to rule out ADHF as a causative etiology. In clinical scenarios where the assessment of acute dyspnea boils down to single or dichotomous pathologies, lung ultrasound can help rule in ADHF. For patients being treated for ADHF, lung ultrasound can also be used to monitor response to therapy. Lung ultrasound is an important adjunct in the management of patients with acute dyspnea or ADHF.

## INTRODUCTION AND BACKGROUND

Lung ultrasound has come a long way the past decade. The old adage that lung tissue is not amenable to ultrasound no longer holds. Previously thought to be a hindrance, artifacts resulting from high attenuation of sound waves by aerated lung are used today to interpret lung pathophysiology. Much of this work must be credited to Lichtenstein *et al* [1], who first pioneered this work. While the technology of ultrasound has continued to advance in terms of image resolution, miniaturization, contrast enhancement, 3D and 4D techniques; lung ultrasound does not require any of the above. In fact, lung ultrasound thrives on the simplest of machines. As manufacturers have sought to reduce or eliminate artifacts that sound produces when attenuated, lung ultrasound has held onto them, requiring the operator to either use older generation machines, or to switch off advanced features on newer machines. 

The most well recognized use of lung ultrasound is in the evaluation of pneumothoraces, where, lung ultrasound has shown clear superiority to the standard chest x-ray [2]. Diagnosis of pneumothorax by thoracic ultrasound is relatively straightforward with consistent findings ruling in or out the disease [3- 5]. In contrast, acute decompensated heart failure (ADHF) is a heterogeneous condition with a broad spectrum of symptomatology and presentations. In the absence of lung ultrasound, the working diagnosis is usually based on history, physical exam, electrocardiography and chest x-ray. However, clinical findings are neither sensitive nor specific [6]. Criteria based diagnostic tools, such as the Framingham Heart Failure criteria [7], while possessing high specificity, lack sensitivity and thus cannot effectively rule out heart failure as a presenting complaint [8]. The chest x-ray and electrocardiograph, while each fairly specific in the presence of certain findings like cardiomegaly or T-wave abnormalities respectively, are also not sensitive enough to preclude the presence of heart failure [6, 9]. The utility of serum testing for NT-proBNP has shown some promise, but remains an area of debate [10]. Definitive diagnosis is reliant on imaging [6, 10]. Echocardiography with Doppler studies is arguably the most important single diagnostic test in evaluating a patient with heart failure [10]. However, formal echocardiography, interpreted by a cardiologist is not always available at the point of care or after hours. Also, in certain clinical scenarios, for example in a patient with chronic heart failure and established echocardiographic findings of poor left ventricular function now presenting with acute pulmonary edema, or in a patient presenting with undifferentiated dyspnea, an immediate full echocardiographic exam may not be indicated. In such instances, lung ultrasound can provide timely data sufficient to support or refute the diagnosis of ADHF.

This review will focus on the available evidence supporting the use of lung ultrasound in the management of acute decompensated heart failure, both as a diagnostic modality and in monitoring heart failure therapy. A literature search was carried out on PubMed/Medline using search terms related to any of the following terms; ultrasound, heart failure, pulmonary edema, dyspnea, and lung. Over three thousand results were retrieved. The title and/or abstract of these results were scanned and high quality articles were selected for full review. Case reports, letters, reviews and editorials were excluded. Articles in which lung ultrasound is used to assess alveolar-interstitial syndrome are presented in Table **1**. Articles with utility of lung ultrasound in acute decompensated heart failure are presented in Table **2**. In the following sections, we will discuss the basic premise of lung ultrasound using the ‘B line’, its link to alveolar-interstitial syndrome, and finally its application in ADHF.

## THE “B-LINE” AND ITS SIGNIFICANCE

**Fig. (1):**
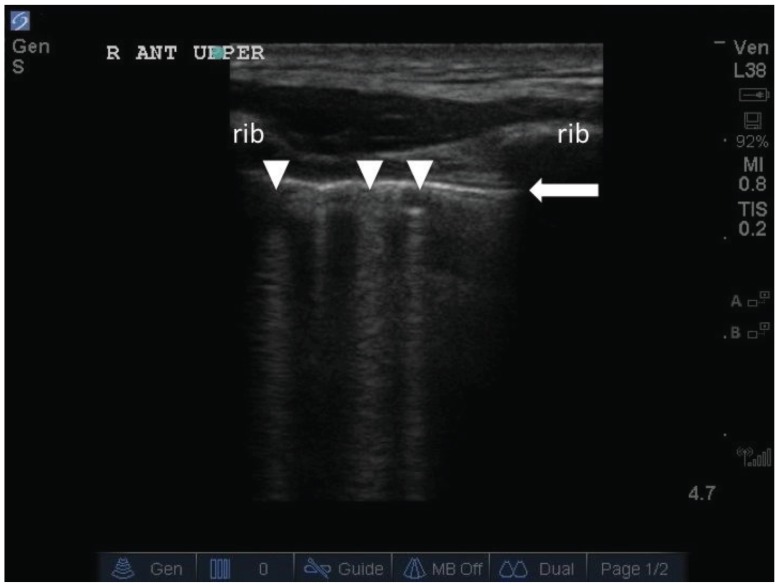
The white arrowheads indicate ***B-lines***. They arise from the pleural line (white arrow), are narrow, and extend vertically to the edge of the screen eliminating any horizontal lines.

**Fig. (2):**
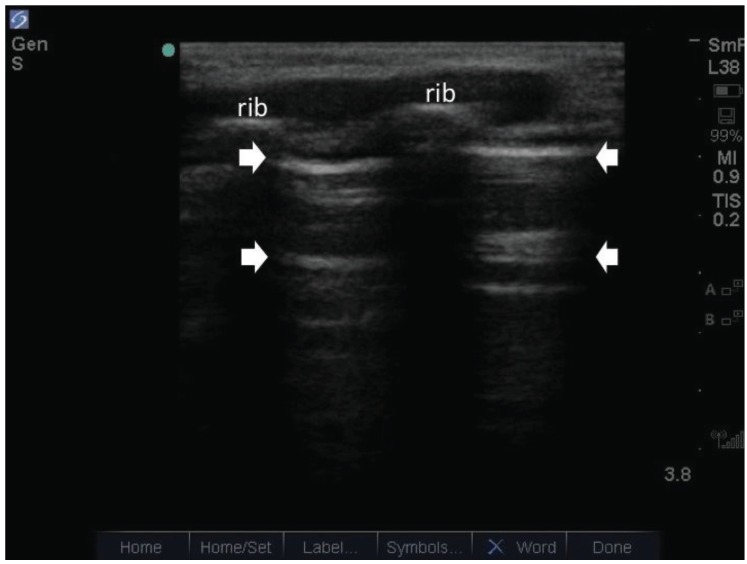
The white arrows indicate horizontal ***A-lines***. They represent reflection artifacts between the pleura and the surface of the chest wall, and are present in normal lung.

A large part of the utility of lung ultrasound is due to the sound artifact known as the “B-line”. Ziskin *et al* first described this artifact in 1982 [11]. B-lines are created from an acoustic impedance mismatch when a sound beam crosses two interfaces, as in between lung and chest wall (Fig. **1**). They arise from the pleural line, appear hyperechoic and extend outwards to the edge of the screen with a narrow fan-like appearance. The B-lines would obliterate any horizontal lines seen below the pleura. These horizontal lines are known as A-lines, which represent reflection artifacts between the pleura and the surface of the chest wall, and are indicative of normal lung (Fig. **2**). The B-line and its link to alveolar-interstitial syndrome (AIS) was first described by Lichtenstein *et al* in his 1997 study [1]. In the study, Lichtenstein *et al* correlated lung ultrasound findings with computerized-tomography (CT) studies in a small subgroup of patients with AIS. The B-line was found to originate from thickened subpleural interlobular septa. It was shown that thickened subpleural interlobular septa each separated with an average distance of 7 +/- 1mm seen on CT, were similar to the average distance between multiple B-lines seen on ultrasound. The alveolar-interstitial syndrome is a clinico-radiographic entity that encompasses a wide range of cardiopulmonary pathologies including both cardiogenic and non-cardiogenic pulmonary edema [12]. Therefore, the relationship between AIS and ADHF can simply be understood as conditions with overlapping clinico-radiographic findings.

In clinical practice however, multiple B-lines do not always follow a regular pattern with equidistant separation of 7 +/- 1mm. Often, multiple B-lines appear closer together, and sometimes coalesce together to form contiguous bands. Soldati *et al* coined the following terms when describing multiple B-lines of increasing severity: (i) “septal syndrome”, an interstitial syndrome where B-lines were separated by the same distance as that of the superficial pleura projections of inter-lobular septa, (ii) “interstitial alveolar syndrome” (aka AIS), where B-lines were separated by a distance less than that in septal syndrome, and (iii) “white lung”, where B-lines completely coalesce [13] (Fig. **3**). This study included a retrospective analysis of the lung ultrasound findings in 136 adult patients. These patients all had radiographic evidence of AIS, had lung CT during the hospital stay, and documentation of the cause of the pulmonary edema. It would appear that regardless of the underlying lung pathology leading to AIS, loss of alveolar volume coupled with edema enriched interstitium account for the reverberation phenomena of B-lines. In this model, cardiogenic pulmonary edema, which originates from edema beginning first within the interstitium before hydrostatic pressures forces water into the alveolar space, would account for diffusely located multiple B-lines that start out separated more or less by the distance between interlobular septa. Pneumogenic interstitial syndrome (e.g. ALI/ARDS), begins in the alveoli due to altered alveolar membrane permeability before involving the interstitium, would account for more closely related B-lines that take root from reverberation artifacts arising from air-water interfaces within the alveoli itself. In the most severe forms, where alveolar flooding occurs, the “white lung” finding is produced. Following Lichtenstein’s work, other authors have also demonstrated good accuracy in using multiple B-lines to diagnose AIS (with sensitivities and specificities from 85-97% and 95-97% respectively) [14-16].

## DEFINING MULTIPLE B-LINES IN POSITIVE LUNG ULTRASOUND

In the current literature, there has not been a strict set of criteria defining how many B-lines constitute a significant lung ultrasound finding. Lichtenstein *et al* first used the presence of at least three B-lines per field of scan seen longitudinally between two ribs, with a distance between two B-lines <7 +/- 1 mm, as criteria for abnormality and most authors have followed this convention [1, 13, 14, 15]. These criteria have been used irrespective of scan technique, whether transverse in the intercostal space or longitudinal across ribs. Usually, a micro-convex or phased array probe with a narrow footprint is used. Some operators choose to use a linear or curvilinear probe with a broad footprint. These probes allow views across several rib spaces, or a longer view of the pleural line. A positive scan with these probes should be similar to the general convention of at least three B-lines less than 7mm apart. 

To comprehensively examine both lungs under ultrasound, several approaches to scan technique have been used. One simple approach is to divide the chest wall into anterior and lateral areas (Fig. **4**) [1, 17]. Another approach divides the chest wall into anterior and lateral portions, upper and lower halves, for four zones per hemithorax [14-18]. The margins delineating the anterior chest are the parasternal and anterior axillary lines, while the lateral chest are defined by the anterior and posterior axillary lines. Yet another approach divides the chest wall into anterior, lateral and posterolateral zones, upper and lower halves, for a total of 6 zones per hemithorax [16]. The posterolateral chest wall is beyond the posterior axillary line and is approached by placing the patient’s arm across the chest plus some minimal movement of the patient. Some authors choose to omit scanning the posterior chest wall; many studies involved scanning critically ill patients in the supine position, and scanning of the anterior and lateral chest are sufficient for the diagnosis of AIS. Regardless of the approach used, patients are scanned in the position of convenience and comfort. Therefore sitting up, semi-recumbent or lying in lateral decubitus may allow imaging of the entire chest. With comprehensive scanning, multiple B-lines may be seen diffusely located across both lungs, or localized to one area of a lung. For the ultrasonographic diagnosis of AIS, the most specific finding is that of multiple B-lines located bilaterally, in either anterior or lateral chests, or in both. When compared to chest x-rays, the above sign gives a specificity of 93-98% and a sensitivity of 85-93% [1, 14, 16]. It should be noted that multiple B-lines could also be observed in the following scenarios: (i) Multiple B-lines are sometimes seen surrounding pneumogenic lesions, e.g. alveolar consolidations like pneumonia. Using lung ultrasound to detect such isolated pulmonary lesions has only a sensitivity of about 64-66% and a specificity of around 63% [1, 15]. (ii) Multiple B-lines are also often seen in the baso-lateral regions of healthy individuals or in patients with normal chest radiographs, occurring in a frequency of around 20-28% [1, 15].

**Fig. (5):**
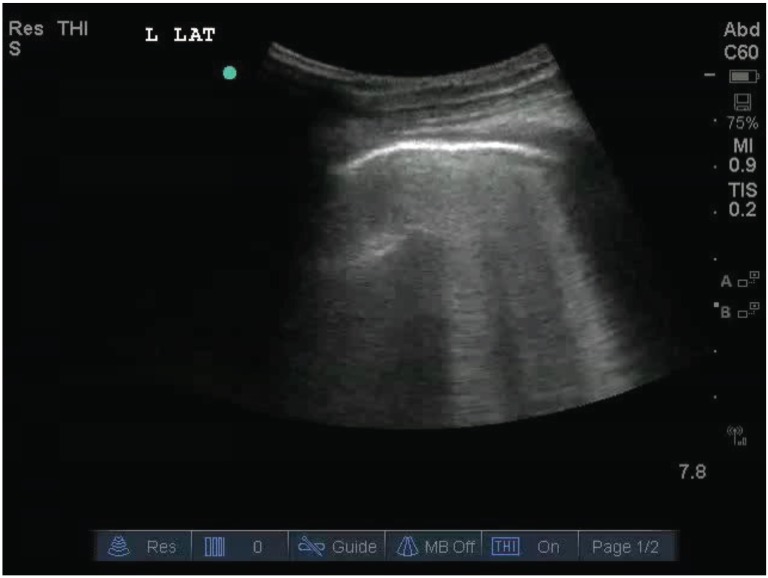
(Video Clip) Reduced lung sliding is seen in a patient with bilateral pneumonia.

As mentioned earlier, AIS describes a clinico-radiographic entity with multiple etiologies. The primary insult may be due to an inflammatory or fibrotic process, or due to an increased extra-vascular lung water load resulting in interstitial followed by alveolar edema. Therefore B-lines are not specific in themselves. How can one use multiple B-lines to differentiate the various causes of AIS? Copetti *et al* sought to answer this question in a study which compared ICU patients who had either acute pulmonary edema (APE) or acute lung injury / acute respiratory distress syndrome (ALI/ARDS) [19]. All patients with APE or ALI/ARDS had diffuse multiple B-lines consistent with the ultrasound diagnosis of AIS. In addition, patients with ALI/ARDS had multiple B-lines that are spread heterogeneously with areas of sparing (normal lung) and areas where B-lines become confluent to form “white lung”. They also had certain pleural ultrasonographic signs like reduction of lung sliding [3] (Fig. **5**, video clip), lung pulse sign [20] (Fig. **6**), and consolidation. None of the patients with APE had these findings, and multiple B-lines tend to be homogeneously distributed. While these specialized lung ultrasound findings hold promise in the evaluation of patients with AIS, there are certain limitations. The ultrasound signs are more advanced and require interpretation by experienced lung operators, the patient cohort reflects a sicker group with more florid signs in the ICU and may not be generalizable to emergency room or general hospital floor patients who may have milder / less florid form of disease.

There were also two studies by Lichtenstein *et al* which compared ultrasonographic multiple B-lines to clinical diagnosis. Both were conducted in the intensive care unit and the authors performed all ultrasound examinations. The first, compared patients diagnosed with cardiogenic pulmonary edema with those with chronic obstructive pulmonary disease exacerbation, along with a control group free of respiratory pathology. Corresponding cardiac or respiratory history, clinical presentation, radiographic and echocardiographic data confirmed diagnoses. Multiple B-lines that were diffusely spread across both anterolateral lungs were 100% sensitive and 92% specific for the diagnosis of pulmonary edema [17]. The second study described and studied the “BLUE protocol” (Bedside Lung Ultrasound in Emergency). Lung ultrasound results were compared with the final diagnosis by the ICU team. The finding of diffuse multiple B-lines was 95% specific and 97% sensitive for cardiogenic pulmonary edema [16]. However, there are some limitations in the two studies. In the first, the study population had diametrically opposed conditions: pulmonary edema (causing AIS) and COPD. In the setting of a patient with undifferentiated dyspnea, lung ultrasound may help elucidate the diagnosis only if the clinical presentation makes those two diagnoses the most likely choices. In the second study, the patient population with bilateral diffuse AIS all had cardiogenic pulmonary edema. The study excluded patients in whom there were overlapping conditions, e.g. COPD with pulmonary edema, pneumonia with pulmonary edema, and there were no patients with ALI/ARDS or chronic interstitial lung disease. Indeed, the authors acknowledged this limitation, which probably explained the overall accuracy of the lung ultrasound protocol at only 90.5%. Despite this, lung ultrasound is useful to the clinician as an adjunct in the diagnostic workup of an acutely dyspneic patient. Bedside ultrasound can quickly lead the clinician to identify and institute rapid treatment in certain conditions with straightforward presentations. The absence of B-lines can also help the clinician effectively rule-out the presence of AIS. 

## LUNG ULTRASOUND COMPARED TO CHEST CT IN THE ASSESSMENT OF AIS

Computerized tomography (CT) of the chest remains the gold standard diagnostic modality for the assessment of lung pathologies like AIS. However, it is not always available, requires transportation and delivers an amount of ionizing radiation that hampers its repeatability. Plain radiography has been shown to perform poorly when assessing AIS [6, 21, 22]. Studies utilizing lung ultrasound to assess AIS involve direct comparison between lung ultrasound findings and plain chest radiographs (Table **1**). To date, there has not been a prospective trial directly comparing lung ultrasound with chest CT. However, in those studies comparing lung ultrasound and chest radiographs, there were subgroups of patients who had chest CT performed and these correlated well with lung ultrasound findings. In Lichtenstein *et al*’s 1997 study, 29/250 patients had chest CT, of which 17/29 patients had AIS. 15 of the 17 patients had diffuse AIS on CT and all of them demonstrated diffuse multiple B-lines [1]. In another study by Volpicelli *et al*, although only 18/300 patients had chest CT performed, all of those patients had lung ultrasound findings that corresponded to CT findings [14]. 

## LUNG ULTRASOUND IN THE ASSESSMENT OF ACUTE DECOMPENSATED HEART FAILURE (ADHF)

Another method for defining lung ultrasound positivity was using the “lung comet score” (Fig. **4**), first described by Jambrik *et al* [23], and subsequently by many other authors in different studies [24-30]. The anterior and lateral chest walls are scanned at fixed locations, along the parasternal, mid-clavicular, anterior axillary and middle axillary lines, from the second to the fifth intercostal space on the right chest, and from the second to the fourth intercostal space on the left chest, for a total of 28 scan sites. Furthermore, the lung comet score can be grouped into grades of severity; “mild” with 5-14 lung comets, “moderate” with 15-29 lung comets and “severe” with >30 lung comets [28]. This score has been used almost exclusively in studies involving heart failure patients. The initial study by Jambrik *et al* investigated 121 patients with varied causes of dyspnea admitted to a cardiology-pulmonology unit [23]. Ultrasound lung comet score for each patient were compared to radiology interpreted chest radiographic extra-vascular lung water (EVLW) score. The study showed significant linear correlation between ultrasonographic lung comet score and radiologic EVLW score (Pearson’s correlation r=0.78, p<0.01). The authors did note a limitation whereby discordant findings between ultrasound and x-ray were due to false-positive lung comet score occurring when x-rays contain other radiologic abnormalities, contributed in part by patients with non-cardiac respiratory conditions. 

In other studies, authors have also demonstrated linear correlations between lung ultrasound comet scores and EVLW. In the first, ultrasound lung comet scores were compared to chest radiography, as well as EVLW and wedge pressure determined invasively using a cardiac output measurement device (PiCCO system), in a small group of 20 patients before and after cardiac surgery [24]. Such a system allows accurate measurements of EVLW, and a normal measured EVLW is less than 500 mLs [31]. The authors also define a positive lung scan as multiple B-lines (at least 3 B-lines <7mm apart) located diffusely across either both antero-lateral or both lateral lung surfaces. The lung comet score had a significant positive correlation with the radiologic EVLW score (r=0.60, p<0.0001) as well as the measured EVLW (r=0.42, p<0.001) and wedge pressure (r=0.48, p<0.001). Also, a positive lung ultrasound is 90% sensitive and 96% specific in determining an EVLW of more than 500 mLs. Likewise, a negative lung ultrasound is 90% sensitive and 89% specific in determining an EVLW of less than 500 mLs. The same authors in a separate study compared lung comet scores between patients with normal cardiac function and those with reduced left ventricular systolic function pre and post exercise, and demonstrated that lung comet scores can detect excess EVLW and correlate well with stress-induced changes in the pulmonary capillary wedge pressure (PCWP) of patients with LV dysfunction [25]. Despite the linear relationship between ultrasound lung comets and measured wedge pressure, the studies described above mainly involved patients with cardiac conditions, and by study design patients with respiratory disorders were excluded. One study investigated 102 ICU patients with heterogeneous diagnoses; where almost 50% of the study cohort were patients with severe sepsis or ARDS [32]. Every patient received pulmonary artery catheterization and lung ultrasound, and the relationship between ultrasonographic AIS and wedge pressure was investigated. Bilateral anterior lung comets, termed “B predominance” by the authors, were seen across a wide range of wedge pressure values. This was not an unexpected finding given the pathophysiology described in the previous section regarding hydrostatic induced cardiogenic pulmonary edema versus permeability induced non-cardiogenic pulmonary edema. In the fourth and largest study, 340 patients admitted to a cardiology service had standard echocardiography as well as lung ultrasound comet score measurements. Patients admitted with acute heart failure had significantly higher lung comet scores, and increasing number of lung comets were seen with increasing severity of dyspnea or NYHA functional classification. Increased lung comet scores were also seen with worsening diastolic dysfunction, and there was a significant inverse relationship between lung comet score and ejection fraction (r= -0.354, p<0.001) [27]. 

Lung comet score has been shown to correlate well with BNP levels. A study of 121 hospitalized patients with dyspnea classified according to NYHA class II to IV had lung ultrasound comet scores as well as serum NT-proBNP levels measurements taken within 4 hours before treatment. Patients’ diagnoses were determined by independent review of clinical data and investigative results. The majority of patients had cardiogenic dyspnea, and they had higher values of lung comet scores as well as BNP when compared to those with non-cardiogenic dyspnea. Lung comet scores were significantly correlated with BNP levels, and a score of four B-lines gave the best diagnostic accuracy with a sensitivity of 81% and a specificity of 85% [26]. A similar study, conducted in the emergency room, recruited 94 patients with acute dyspnea and compared lung ultrasound alone, compared to BNP levels, as well as combined with BNP levels in the diagnosis of ADHF [18]. Instead of the lung comet score, the authors divided each lung into four zones for scanning (described above, also see Fig. **4**). A positive test was defined as two positive zones on each side. A positive finding, especially if it includes both lower lateral zones, enables lung ultrasound to predict ADHF with equal performance to BNP. Also, an eight zones ultrasound that is completely positive or negative had the greatest predictive value in diagnosing or dispelling ADHF (a positive eight zones ultrasound has infinite LR, a negative 8 zones ultrasound has LR = 0.22 with 95% CI = 0.06 to 0.80). 

So far, the focus has been on the detection of B-lines as a marker of lung congestion in ADHF. Another sign of congestion or lung fluid accumulation is pleural effusion, which is a common finding in heart failure. Some authors have asked the question if lung ultrasound could use pleural effusion detection as a marker for ADHF. In one study, 60 patients with ADHF confirmed by clinical findings, echocardiography and chest CT, had lung ultrasound performed to detect pleural effusion (echocardiography and lung ultrasound were performed by two clinicians, presumably the authors). A small group of 20 patients without respiratory or cardiovascular disease acted as controls. Incidence of pleural effusion in the group of heart failure patients was 91%. Lung ultrasound had a sensitivity of 90% and a specificity of 95% in detecting pleural effusions, compared to chest x-ray which had a sensitivity of only 43% though specificity was a good 100% [33]. In a separate study, the authors also followed up 46 heart failure patients with a history of prior decompensation for over two years. 26 of these patients had an episode of decompensation, and pleural effusion was the most frequent sign observed during decompensation. In this cohort, when compared to BNP levels as a reference standard for decompensation, lung ultrasound had a sensitivity of 74%, negative predictive value of 73%, and accuracy of 78% for detecting decompensated heart failure [34]. Regardless, for the detection of pleural effusions, lung ultrasound is highly accurate and performs much better than chest radiology [21, 35, 36]. 

To improve diagnostic accuracy of lung ultrasound in assessing an acute dyspneic patient for ADHF, it might be possible to combine ultrasound findings of multiple B-lines or lung comet score with pleural effusion. With ADHF, excess lung water is likely to occur as interstitial edema or pleural effusions or both, though they may be mutually exclusive. To date, there has not yet been a study that uses both ultrasonographic signs to diagnose ADHF. Furthermore, the performance of lung ultrasound using multiple B-lines or the lung comet score seems akin to that of BNP. Multiple studies on BNP has shown its diagnostic accuracy in ruling out heart failure as a cause of acute dyspnea when the serum level is low, and heart failure is a likely cause when the serum level is high [6, 37, 38, 39]. In a similar fashion, lung ultrasound can effectively rule out pulmonary edema when there is an absence of multiple B-lines or a low lung comet score (<5), and pulmonary edema becomes highly suggestive when there is diffuse widespread lung comets seen. Again, when BNP levels lie in the intermediate grey zone where its diagnostic ability is reduced and a large dose of clinical correlation is required, lung ultrasound also suffers the same drawback. At least, lung ultrasound can be quickly performed at the bedside with little to no turnaround time and lead to rapid therapeutic decisions. This is ultrasound’s strength, especially in critical care settings such as the emergency room or the intensive care unit. 

## LUNG ULTRASOUND IN ACUTE DECOMPENSATED HEART FAILURE THERAPY

The adequacy of therapy in ADHF is usually assessed clinically; by resolution of symptoms and clinical signs of pulmonary congestion like rales. Chest radiography is the standard for assessing signs of lung water excess but it is well known that patients with acute pulmonary edema may lack radiographic signs at presentation and these signs may lag behind the clinical picture by as much as 6 hours [40]. Lung ultrasound can readily identify EVLW by assessing multiple B-lines or lung comet scores and it can potentially assist the clinician in monitoring the adequacy of heart failure treatment. So far, three studies have shown how diuresis has resulted in the disappearance of multiple B-lines. 

This first study recruited patients with ADHF, and compared them at presentation and at the end of hospital stay after treatment. Lung ultrasound, chest x-ray, physical exam, serum BNP were performed at presentation and repeated at discharge. The authors found a significant clearing of the B-lines at discharge [41]. Two other studies also showed significant reduction in B-lines when comparing patients before and after hemodialysis [29, 30]. In the study by Noble *et al*, 40 patients had lung ultrasound performed before, mid-way and after hemodialysis and the lung comet score was correlated to the volume of fluid removed which was calculated by the dialysis machine.

In the study by Mallamaci *et al*, patients were classified as symptomatic or asymptomatic with a modified NYHA classification, and total body water was estimated with bioelectrical impedance analysis. Lung ultrasound and echocardiography were performed before and after hemodialysis in all patients. While these studies demonstrated significant reductions in B-lines after dialysis or treatment, some interesting observations can be made. Patients with worse cardiac function or lower ejection fraction had more B-lines pre-dialysis, and lung water excess appears to be strongly associated with a poorer ejection fraction rather than total body water estimates. The clearance of B-lines appears to occur in real time and could allow physicians to monitor and taper treatment accordingly. The reduction in B-lines occur in line with either radiographic or clinical findings, but not with BNP levels, suggesting that serum BNP may not reliably indicate pulmonary congestion being resolved. Also, the clearance of ultrasonographic B-lines appears unrelated to subjective dyspnea scores [29], and a majority of asymptomatic patients had at least a moderate lung comet score [30]. These facts suggest that presence of B-lines may be a useful indicator to detect patients in pre-clinical heart failure. 

Finally, Frassi *et al* showed how ultrasound lung comets could be a prognostic indicator on par with ejection fraction measured by echocardiography. The study followed 290 in-hospital patients admitted to a cardio-pulmonary unit, the majority of whom had coronary artery disease, for a median period of 16 months. Echocardiography and lung ultrasound for lung comet score were performed on all patients, by a cardiologist and a trained ultrasonographer. Patients with a severe grade of lung comet scores (>30) had a worse outcome including repeat hospitalization [28].

## IS LUNG ULTRASOUND READY FOR PRIME TIME USE?

1. There is no standard technique to examine the lungs ultrasonographically. From the described literature, scan techniques described can be broadly divided into two groups: scanning by zones or scanning by fixed points with a cumulative lung comet score. The three zones described by Lichtenstein *et al* is easiest to use, most practical and most widely used [16]. The lung comet score is useful in monitoring heart failure therapy as described by the above studies [29, 41], but in reality an overall impression of B line quantity may very well be sufficient for clinical practice. More studies in this area utilizing a zonal approach to lung scanning are warranted.
2. When only single pathologies are in the differential, for example acute cardiogenic pulmonary edema versus exacerbation of chronic obstructive pulmonary disease, lung ultrasound can be used alone to accurately rule out pulmonary edema. When the presenting patient has features suggesting a high likelihood of ADHF, lung ultrasound may be used to rule in cardiogenic pulmonary edema. Howevere, there is only one small study that examines ultrasound features differentiating AIS caused by ALI/ARDS from cardiogenic pulmonary edema [19]. This is probably the achilles heel of lung ultrasound in the assessment of ADHF. In the setting of a sick heterogeneous patient population with multiple co-morbidities and diagnoses, lung ultrasound might not be able to distinguish cardiogenic pulmonary edema from other lung pathology associated with AIS. In such situations, lung ultrasound should be used only as an adjunct to the overall clinical impression.
3. The majority of studies had lung ultrasound performed by experienced operators. The ultrasound exam was performed within 10 minutes, and many studies claim completion of the exam within 3-5 minutes. Real world applicability may not see such rapid exam times amongst operators with lesser experience. While B-lines are not technically difficult to interpret even by novice performers with minimal training [42], a more comprehensive suite of lung ultrasound findings including pleural line and parenchymal abnormalities will require some form of training program. A reporting protocol should also be implemented to ensure uniformity, completeness and accuracy for all performers of lung ultrasound within a service or hospital [43].

## CONCLUSION

The greatest utility of lung ultrasound is in the management of critically ill patients, for whom trasnsportation off the unit for formal diagnostic imaging may be dangerous. Ultrasound machines are more portable than ever and ubiquitous in emergency departments, operating rooms and intensive care units. The providers managing the patient are able to perform the required study and interpret the findings themselves, lending weight to the clinical picture. In the management of ADHF, lung ultrasound has clearly shown utility as an aid to diagnosis and in monitoring therapy. Although unlikely to replace chest radiography in the short term, the role of lung ultrasound in ADHF will undoubtedly grow more common with time to the great benefit of our patients.

## Figures and Tables

**Fig. (3). (a):**
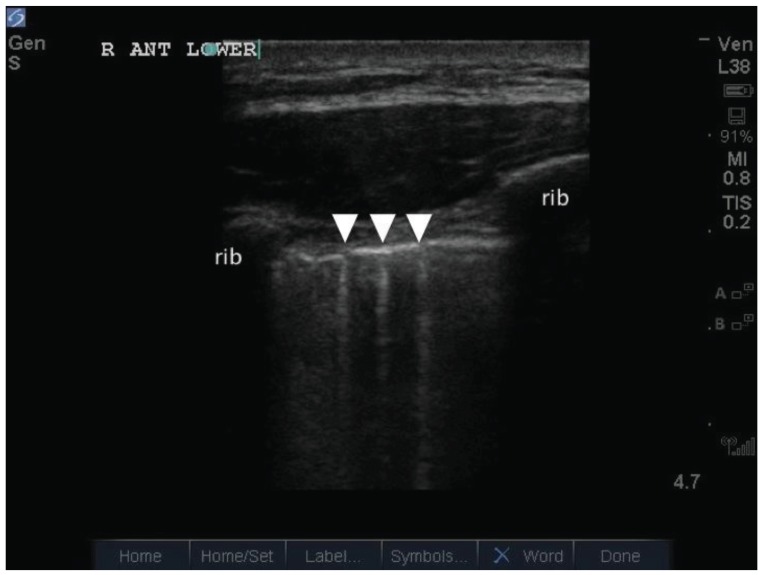
Multiple ***B-lines*** with equidistant separation of 7 mm, seen in septal syndrome.

**Fig. (3). (b):**
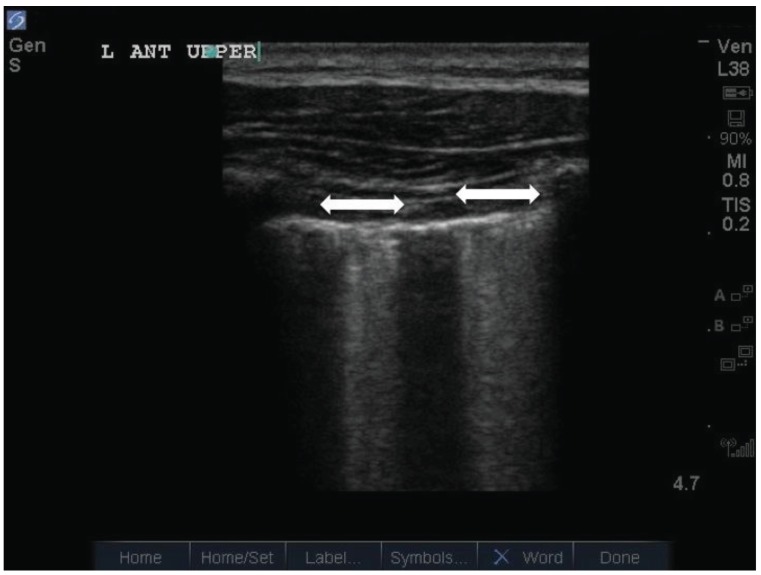
Confluence of multiple B lines, appearing as two distinct bands outlined by the white double-headed arrows.

**Fig. (3). (c):**
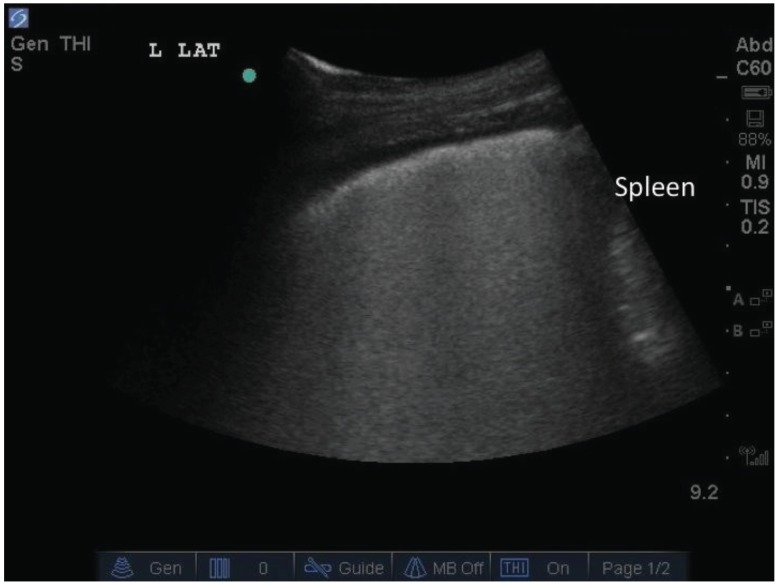
Confluence of multiple B lines into what appears as the hyperechoic ‘white lung’.

**Fig. (4). (a):**
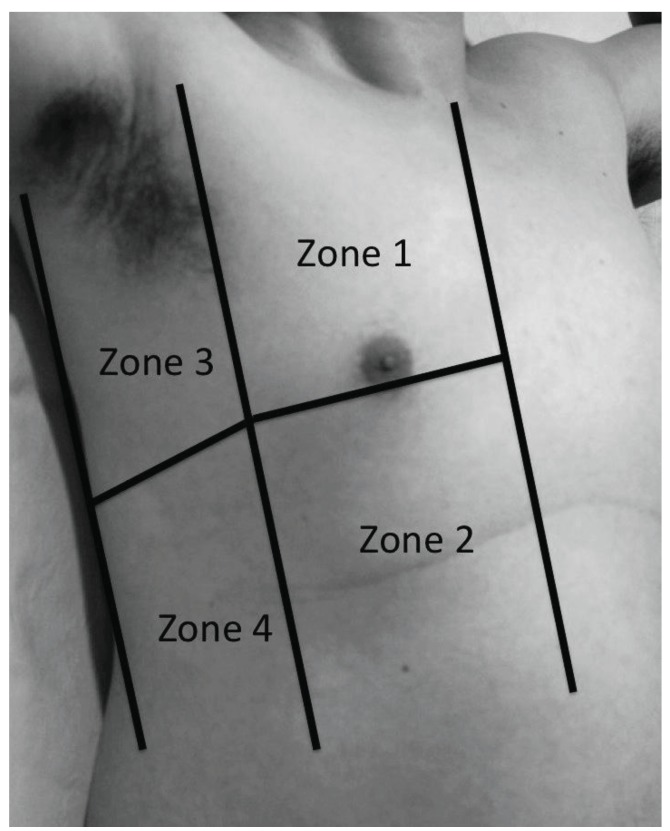
The right chest wall is divided into anterior and lateral, upper and lower halves for four zones per side. The margins delineating the anterior chest are the parasternal and anterior axillary lines, while the lateral chest are defined by the anterior and posterior axillary lines.

**Fig. (4). (b):**
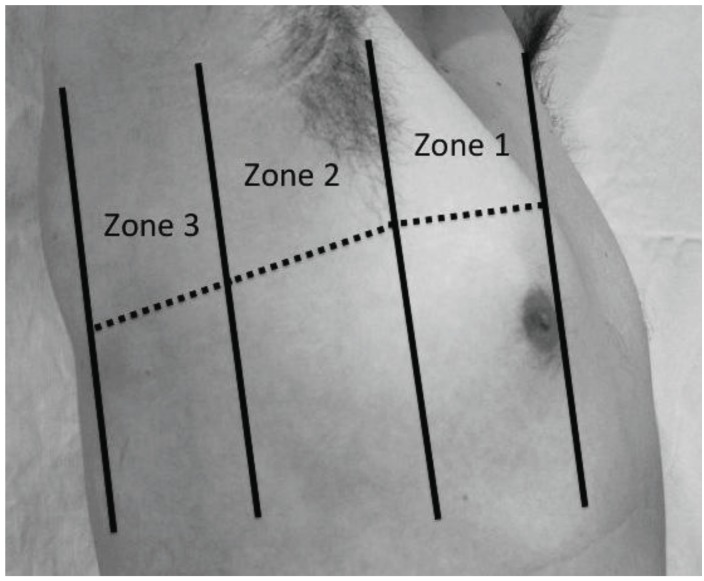
The right chest is divided into anterior, lateral and posterolateral zones. Again, this can be further subdivided into upper and lower halves for a total of six zones per side. The patient is usually slightly turned to expose zone 3, which is beyond the posterior axillary line.

**Fig. (4). (c):**
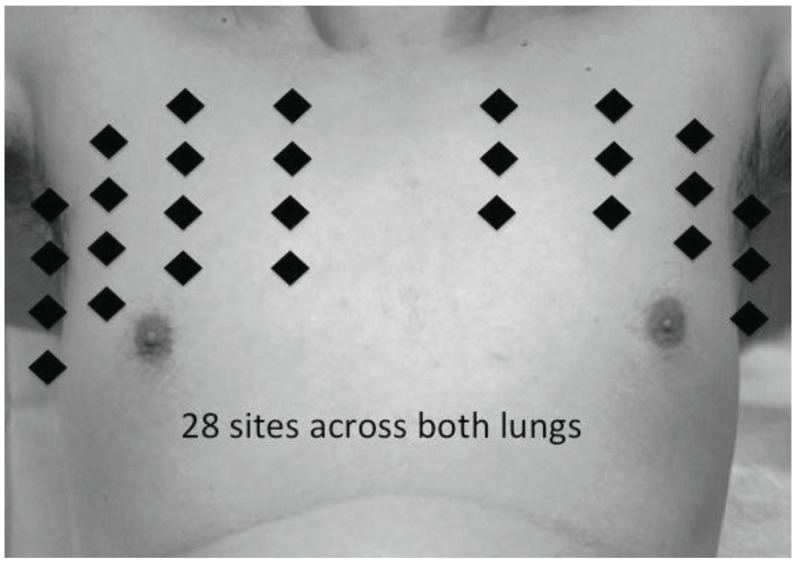
Fixed locations on the anterior and lateral chest walls are placed along the parasternal, mid-clavicular, anterior axillary and middle axillary lines, from the second to the fifth intercostal space on the right chest, and from the second to the fourth intercostal space on the left chest, for a total of 28 scan sites.

**Fig. (6a):**
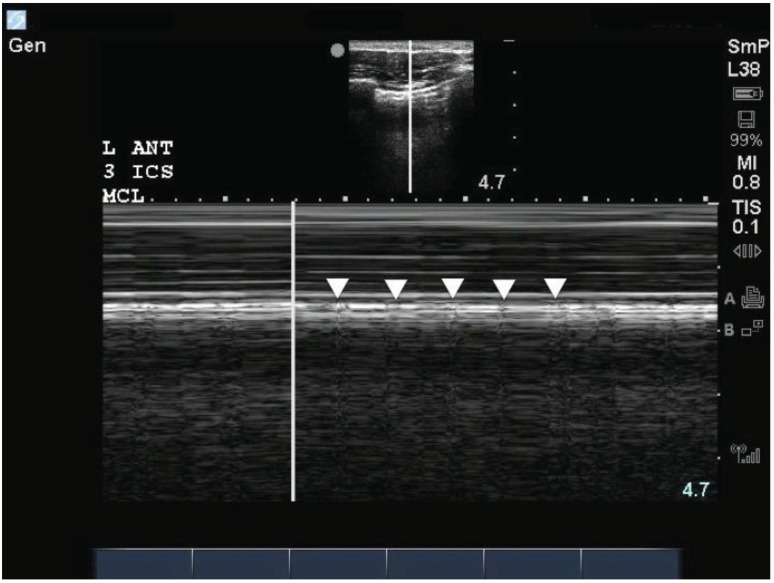
With M mode ultrasound, regular small movements (white arrowheads) can be seen under conditions with reduced lung sliding. These are known as lung pulses, which are due to tiny movements between the parietal and visceral pleura created by transmission of cardiac pulsations to the lung.

**Fig. (6b):**
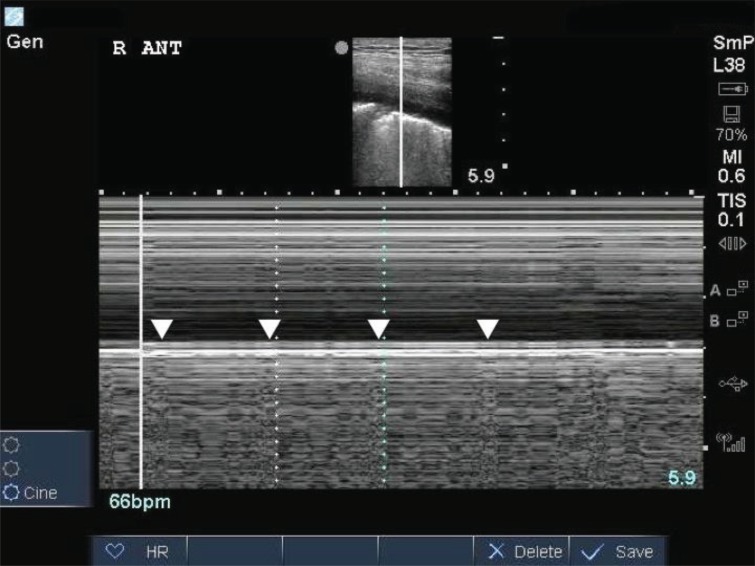
The heart rate can be calculated by measuring the intervals between the lung pulses.

**Table 1.** Studies Where Lung Ultrasound is Used in the Assessment of AIS

Study	Methodology	Ultrasound technique	Results / Comments
Lichtenstein *et al* 1997 [1]	129 patients with AIS on CXR vs 121 patients without AIS. Lung ultrasound compared to CXR with CT correlation in some discordant cases. Setting: ICU US operator: single experienced clinician	Longitudinal scans of anterior and lateral chest walls of patients in supine position. 1st described pathologic pattern as multiple B-lines (at least three) less than 7mm between two rib spaces. Positive scan defined as above pattern observed diffusely over entire lungs, over anterior or lateral lungs only, or patchy.	For diagnosing AIS, positive lung ultrasound has: Sensitivity 93.4% Specificity 93% 28% of patients with normal CXR had baso-lateral multiple B-lines.
Volpicelli *et al* 2006 [14]	300 patients. 160 had cardiopulmonary conditions, of which 75 had AIS. Lung ultrasound compared to CXR (US performed within 48 hrs). CT correlation in 18 cases. Setting: ED US operator: 5 specially trained operators (3 emergency physicians and 2 radiologists)	Scanning of anterior and lateral chest walls, patient in supine position. Each chest divided into four zones (anterior & lateral, upper & lower). Positive scan defined as: pathologic pattern found in >1 zone on each side, both sides involved.	For diagnosing AIS (vs CXR): Sensitivity 85.7% Specificity 97.7% For diagnosing AIS (vs CXR): Sensitivity 85.3% Specificity 96.8% All CT findings correlated to US.
Volpicelli *et al* 2008 [15]	217 patients without clinical or radiologic AIS. Lung ultrasound compared to CXR. Setting: ED US operator: 5 specially trained operators (3 emergency physicians and 2 radiologists)	Scanning of anterior and lateral chest walls, patient in supine position. Each chest divided into four zones (anterior & lateral, upper & lower).	13.2% of scans positive for multiple B-lines. A positive scan was more likely due to isolated alveolar consolidations or baso-lateral regions.
Lichtenstein *et al* 2008 [16]	301 consecutive ICU patients with acute respiratory failure. Lung ultrasound compared with final ICU diagnosis. Setting: ICU US operator: 2 experienced clinicians	Longitudinal scans of the anterior and lateral chest wall, divided into three zones, each divided into upper and lower halves. (six zones total)supine position.	Diffuse bilateral multiple B-lines had 95% specificity and 97% sensitivity for diagnosing cardiogenic pulmonary edema. Patients with chronic interstitial lung disease, mixed diagnosis (e.g. pulmonary edema with COPD, pneumonia with pulmonary edema etc) and patients with unknown diagnosis excluded.
Copetti *et al* 2008 [19]	58 patients fulfilling criteria for either ALI/ARDS (18) or APE (40). Lung ultrasound compared to clinical and radiologic diagnosis. Setting: ICU US operator: 2 experienced clinicians	Longitudinal and transverse scanning of anterior chest (two zones), lateral chest (two zones) and posterior chest. Supine, lateral or seated patient positions.	All patients had sonographic AIS. Reduced lung sliding, lung pulse, consolidation and spared areas were seen only in patients with ALI/ARDS. Pleural effusions were common, 66.6% of ALI/ARDS and 95% of APE patients.
Soldati *et al* 2009 [13]	Retrospective analysis of 176 inpatients with clinical and radiographic evidence of AIS. Ultrasound compared to CXR. CT performed in all patients except neonates. Setting: ED and neonatal unit US operator: 2 experienced clinicians	Transverse scanning across anatomic thoracic lines in each intercostal space.	B-lines occur in a spectrum of severity from a distance separated by interlobular septa (seen only in some cases of cardiogenic pulmonary edema), to confluence in white lung (seen in both ARDS and APE)

**Table 2.** Studies Where Lung Ultrasound is used to Assess Diagnose and Manage ADHF

Study	Methodology	Ultrasound Technique	Results / Comments
Lichtenstein *et al* 1998 [17]	146 patients: 40 with cardiogenic pulmonary edema, 26 with COPD exacerbation, 80 patients without respiratory disorder. Lung ultrasound compared to CXR. Setting: ICU US operator: single experienced clinician	Longitudinal scans of anterior and lateral chest walls of patients in semi-recumbent position. Positive test defined as bilateral multiple B-lines diffuse anterolateral or lateral.	Positive lung US has: Sensitivity 100% Specificity 92% (For diagnosing cardiogenic pulmonary edema)
Kataoka *et al* 2000 [33]	60 patients admitted for ADHF. Lung US compared to clinical variables, CXR, thoracic CT, for diagnosis of pleural effusions. Setting: inpatients US operator: 2 experienced clinicians	Seated patient, scanning through liver and spleen as acoustic windows through rib spaces to detect pleural effusion.	Ultrasound identified 92% and 93% of pleural effusions in R and L lungs respectively compared to 48% and 26% for CXR.
Jambrik *et al* 2004 [23]	121 patients. Lung US compared to CXR for assessment of EVLW. Setting: inpatients (cardiac/pulmonary unit) US operator: experienced clinicians	Lung comet score. 1st to describe scanning in 28 fixed sites over both lungs, corresponding to anatomic thoracic lines in each intercostal space	Significant linear correlation between lung comet score and radiologic lung water score, even stronger correlation in intrapatient variations
Agricola *et al* 2005 [24]	20 patients. Lung ultrasound compared to CXR, PiCCO, for assessment of EVLW. Setting: inpatients pre and post cardiac surgery US operator: 2 experienced clinicians	Lung comet score (as above)	Significant linear correlation between lung comet score and EVLW determined by PiCCO, as well as radiologic lung water score.
Agricola *et al* 2006 [25]	72 patients, 53 with EF<40%, 19 with normal LV function. Lung US to assess variations in EVLW, compared to 2D echo indices of LV function, pre & post exercise. Setting: presumed inpatients >US operator: presumed 2 experienced clinicians	Lung comet score (as above)	Lung comets can assess excess EVLW and its variation during exercise.
Kataoka *et al* 2007 [34]	46 patients with history of ADHF followed over 2.5 years. US detected pleural effusion as a marker for HF decompensation, compared to clinical findings, and using BNP as reference standard. Setting: ED/outpatient consult patients US operator: single clinician	Patient seated, scanned posteriorly along paravertebral, scapular, and posterior axillary lines.	lung ultrasound has sensitivity of 74%, negative predictive value of 73%, and predictive accuracy of 78%, for identifying patients with HF decompensation.
Frassi *et al* 2007 [27]	340 patients. Lung ultrasound and 2D echo correlation. Setting: inpatients of a cardiology-pneumology unit US operator: Experienced cardiologist and trained ultrasonographer	Lung comet score (as above)	Increase lung comet score associated with LV dysfunction, reduced EF and worse NYHA class.
Frassi *et al* 2007 [28]	290 patients admitted for dyspnea and/or chest pain syndrome followed up for median of 16 months. Lung ultrasound and 2D echo compared for prognostication value Setting: inpatients of a cardiology-pneumology unit US operator: unspecified experienced operators, presumed similar to above	Lung comet score (as above) Grading of lung comet score into: Mild: 5-14 comets Moderate: 15-29 comets Severe: >30 comets	Severe lung comet score grade associated with a worse outcome. The higher the score, the worse the outcome.
Gargani *et al* 2008 [26]	149 patients with acute dyspnea, of which 122 were due to CHF. Lung US compared to BNP for the diagnosis of cardiogenic pulmonary edema. Setting: inpatients of a cardiology-pneumology unit US operator: unspecified experienced operators, presumed similar to above	Lung comet score (as above)	Lung comet scores significantly correlated with BNP levels. Lung ultrasound with lung comet scoring has an overall sensitivity of 76% and specificity of 88.8 % for diagnosing pulmonary edema.
Liteplo *et al* 2008 [18]	100 patients with dyspnea. Lung US compared to BNP for diagnosing CHF. Setting: ED patients US operator: experienced emergency physicians or specially trained medical students	Scanning of anterior and lateral chest walls, patient in supine position. Each chest divided into four zones (anterior & lateral, upper & lower). Positive scan defined as: pathologic pattern found in >1 zone on each side, both sides involved.	Eight zones positive or negative lung US had strongest predictive value for ruling in/out CHF. Two zones positive (lower lateral) lung US had moderate predictive value.
Volpicelli *et al* 2008 [41]	81 patients with confirmed ADHF. Lung US, CXR, clinical and BNP compared on admission and at discharge. Setting: ED patients US Operator: experienced clinicians	Longitudinal scans of supine patients with chest divided into 11 areas (three anterior and three lateral on right side and two anterior and three lateral on left side), giving rise to a score 0-11.	Sonographic score correlated with radiologic, clinical scores and BNP at admission. There is significant clearing of sonographic scores after treatment. Change in sonographic score correlated with change in radiologic and clinical scores.
Noble *et al* 2009 [29]	40 patients. Lung ultrasound, and dyspnea scores compared before, during and after dialysis. Setting: inpatients undergoing hemodialysis US operator: experienced clinicians	Lung comet score (as above)	Statistically significant reductions in B-lines from predialysis to midpoint to end.
Lichtenstein *et al* 2009 [32]	102 patients, intubated, with PAC inserted. Relationship between lung US detected AIS and wedge pressure. Setting: ICU patients US operator: 2 experienced clinicians	Longitudinal scanning of anterior chest upper or lower points, or dividing the anterior chest into four zones.	Multiple B-lines can be observed in wide range of wedge pressure.
Mallamaci *et al* 2010 [30]	75 patients undergoing hemodialysis. Lung ultrasound, 2D echo performed before and after. Total body water estimated by bioelectrical impedance analysis. Setting: Inpatient dialysis unit US Operator: experienced technician and novice clinician with limited training	Lung comet score (as above) Grading of lung comet score into: Mild: 5-14 comets Moderate: 15-29 comets Severe: >30 comets	Excess EVLW strongly associated with LVEF, both before and after dialysis. Lung water excess was unrelated to total body water excess.
Zanobetti *et al* [35]	404 patients, all had US and CXR performed. Chest CT done when there is mismatch between CXR and US. Setting: ED patients US operator: single experienced ED physicia	Supine patient, longitudinal and transversal scanning of both hemithoraces along the parasternal, midclavicular, anterior, middle and posterior axillary lines. Then, in sitting position, scanning along the posterior paravertebral lines.	CXR and US had high concordance especially when there is pulmonary edema (kappa = 95%).
